# An Integrated Pan-Cancer Analysis and Structure-Based Virtual Screening of GPR15

**DOI:** 10.3390/ijms20246226

**Published:** 2019-12-10

**Authors:** Yanjing Wang, Xiangeng Wang, Yi Xiong, Cheng-Dong Li, Qin Xu, Lu Shen, Aman Chandra Kaushik, Dong-Qing Wei

**Affiliations:** 1 State Key Laboratory of Microbial Metabolism, School of Life Sciences and Biotechnology, and Joint Laboratory of International Cooperation in Metabolic and Developmental Sciences, Ministry of Education, Shanghai Jiao Tong University, Shanghai 200240, China; wangyanjing@sjtu.edu.cn (Y.W.); wangxiangeng@sjtu.edu.cn (X.W.); xiongyi@sjtu.edu.cn (Y.X.); lcd805728463@sjtu.edu.cn (C.-D.L.); xuqin523@foxmail.com (Q.X.); 2 Bio-X Institutes, Key Laboratory for the Genetics of Developmental and Neuropsychiatric Disorders, Ministry of Education, Shanghai Jiao Tong University, Shanghai 200030, China; yoyomailer@sjtu.edu.cn; 3 Wuxi School of Medicine, Jiangnan University, Wuxi 214122, China; 4 Peng Cheng Laboratory, Vanke Cloud City Phase I Building 8, Xili Street, Nanshan District, Shenzhen 518055, China

**Keywords:** Orphan receptor *GPR15/BOB*, pan-cancer, TCGA, cancer immunity, differential gene expression, prognosis, virtual screening

## Abstract

G protein-coupled receptor 15 (GPR15, also known as BOB) is an extensively studied orphan G protein-coupled receptors (GPCRs) involving human immunodeficiency virus (HIV) infection, colonic inflammation, and smoking-related diseases. Recently, GPR15 was deorphanized and its corresponding natural ligand demonstrated an ability to inhibit cancer cell growth. However, no study reported the potential role of GPR15 in a pan-cancer manner. Using large-scale publicly available data from the Cancer Genome Atlas (TCGA) and the Genotype-Tissue Expression (GTEx) databases, we found that *GPR15* expression is significantly lower in colon adenocarcinoma (COAD) and rectal adenocarcinoma (READ) than in normal tissues. Among 33 cancer types, *GPR15* expression was significantly positively correlated with the prognoses of COAD, neck squamous carcinoma (HNSC), and lung adenocarcinoma (LUAD) and significantly negatively correlated with stomach adenocarcinoma (STAD). This study also revealed that commonly upregulated gene sets in the high GPR15 expression group (stratified via median) of COAD, HNSC, LUAD, and STAD are enriched in immune systems, indicating that GPR15 might be considered as a potential target for cancer immunotherapy. Furthermore, we modelled the 3D structure of GPR15 and conducted structure-based virtual screening. The top eight hit compounds were screened and then subjected to molecular dynamics (MD) simulation for stability analysis. Our study provides novel insights into the role of GPR15 in a pan-cancer manner and discovered a potential hit compound for GPR15 antagonists.

## 1. Introduction

G protein-coupled receptors (GPCRs), also known as seven-transmembrane domain receptors, constitute the largest family of cell signaling receptors [1]. GPCRs respond to a wide range of extracellular signals and regulate various cellular and physiological processes, including hormone regulation, vision, immune responses, neuronal communication, and behavior [2]. Overwhelming evidences have demonstrated that GPCRs and their downstream signaling targets play critical roles in cancer initiation and progression by regulating signal transduction and cellular processes (including cell proliferation, apoptosis, stress signals, immune escape, invasion, angiogenesis and metastasis, ion and nutrient transport and migration) [3,4]. It has also been demonstrated that diverse GPCRs were overexpressed in a variety of tumors [5]. Both orphan and well-characterized GPCRs have been reported to be involved in cancer development [6,7,8,9,10], which provide opportunities for the development of new strategies of cancer prevention and treatment. At present, GPCR-targeted drugs for cancer treatment are still few. The limited concrete knowledge about the role of GPCRs in cancers might be the cause of this lack of GPCR-targeted drugs as a treatment for cancer.

G protein-coupled receptor 15 (GPR15, also known as BOB) is an extensively studied orphan GPCR [11]. It is a chemokine co-receptor of human immunodeficiency virus type 1 and 2 [12] and a meditator of homing control in the large intestine and skin [8]. Dozens of studies have demonstrated the significant association between GPR15 and the immune system. For example, GPR15 was found to be expressed in memory B cells, plasmablasts, and regulatory T cell subsets [13,14]. It directs T cell homing to the developing epidermis as well as to the colon and regulates colitis [8,13,15,16]. When GPR15 controls the homing of FOXP3+ regulatory T cells (T_regs_) to the large intestine lamina propria, it alleviates colonic inflammation [8]. Mounting evidences have suggested that inflammation may help tumor cells to evade the defense from the immune system [17]. The altered expression level and epigenetic regulation of GPR15 could also have a significant influence in the health status of smokers [18,19,20]. Moreover, recent studies reported that GPR15 was deorphanized and its ligand can also bind to SUSD2. The co-expression pattern of GPR15L and SUSD2 can suppress proliferation of several tumoral cell lines via G1 arrest [21,22,23]. This finding indicated that GPR15 may be actively involved in cancer progression. Therefore, it is necessary to characterize the role of GPR15 in carcinogenesis. 

In this study, we firstly performed pan-cancer analysis to elucidate the potential role of GPR15 in cancers. Earlier studies using similar methods have been published to provide new insights of specific genes in carcinogenesis [24,25,26,27,28]. The expression levels of GPR15 were evaluated in 33 different cancers using the data from the Cancer Genome Atlas (TCGA) and the Genotype-Tissue Expression (GTEx) databases. The function of GPR15 was predicted by integrated network analysis. Our study identified a number of common genes that are in the GPR15 regulatory network in four cancers. We provide evidence that GPR15 acts as an immunomodulator and can be considered as a novel target for immunotherapy for the four cancers. We also predicted the 3D structure of human GPR15 and applied structure-based virtual screening (SBVS) approaches [29,30,31,32,33,34,35,36,37] to discover potential antagonists that bind to the predicted active site. These results help us to understand the role of GPR15 in carcinogenesis and its future prospective for STAD drug development. 

## 2. Results

### 2.1. Pan-Cancer Mutational and Expression Landscape of GPR15

**Figure 1:**
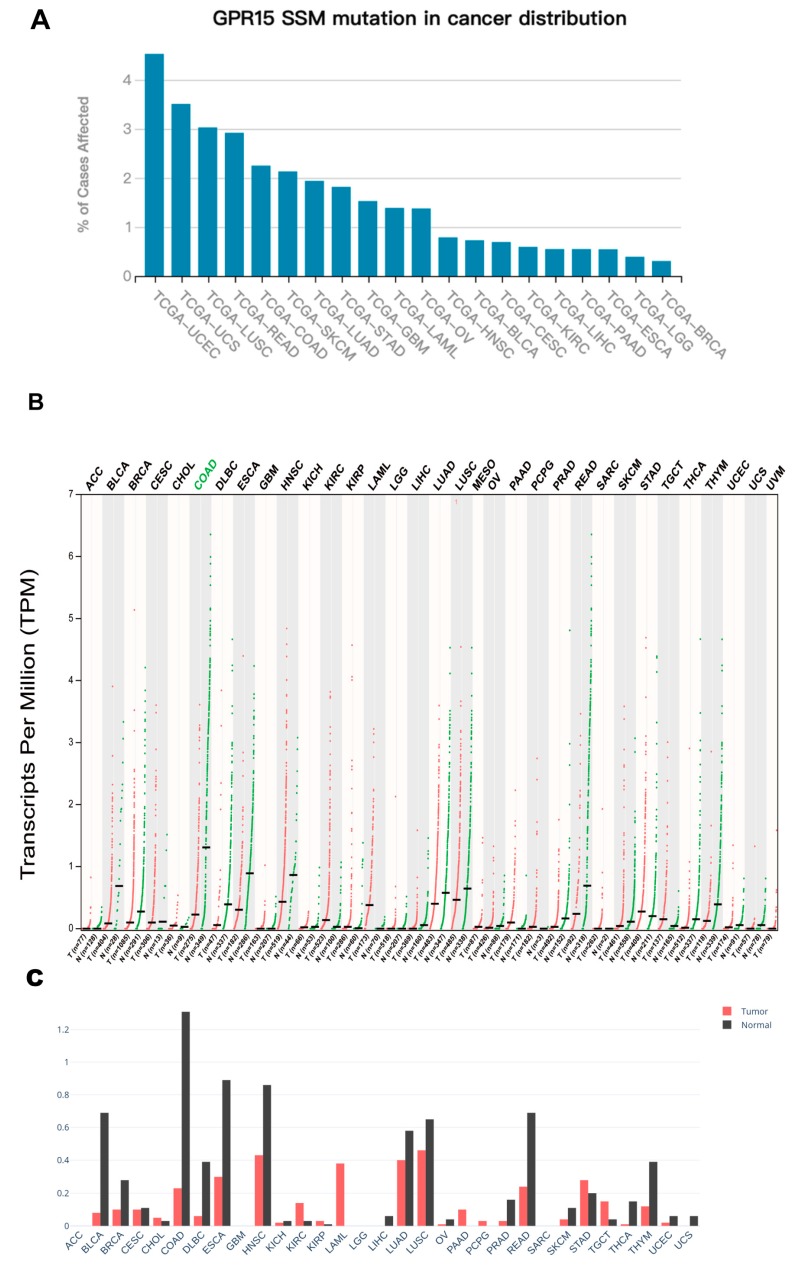
Expression and mutational landscape of GPR15 in the Cancer Genome Atlas (TCGA) cohorts. (**A**). Y-axis represents mutational rates of GPR15 (simple somatic mutation) in all TCGA cohorts. The cancer types whose GPR15 mutational rate is 0 are excluded. (**B**)**.** Pan-cancer expression landscape of GPR15. “T” stands for tumor tissue and “N” stands for paired normal tissue. The expression abundance is measured by log-normalized transcripts per million (TPM). The green color of the cancer type means that GPR15 is differentially expressed between tumor tissue and paired normal cell. (**C**) Bar graph of the gene expression profile across all tumor samples and paired normal tissues. The height of bar represents the median expression (log-normalized TPM) of certain tumor type or normal tissue.

Among all of the 33 cancer types from the TCGA database, cancers with significant differential GPR15 expression (on the basis of difference of median expression between cancer samples and paired normal samples) are COAD (downregulated, *p* = 3.06 × 10^−12^) and READ (downregulated, *p* = 6.80 × 10^−4^) (Figure 1C, Figure S1). Also, COAD showed significantly lower expression in tumor tissue compared to healthy tissues from the Genotype-Tissue Expression (GTEx) project. The expression landscape of GPR15 in TCGA cohorts is shown in Figure 1B. 

**Figure 2:**
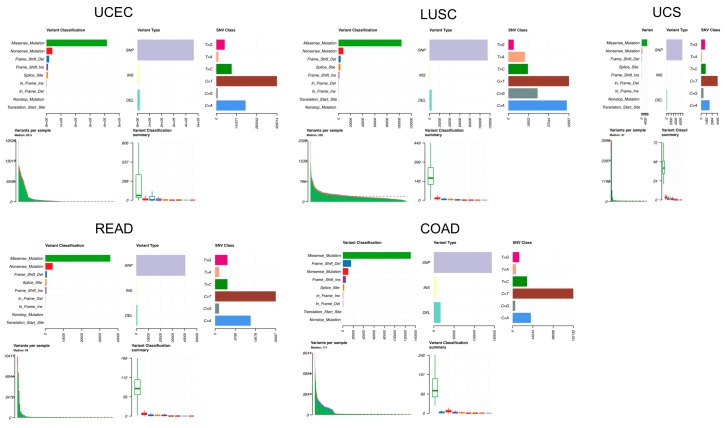
Mutational summary plot of uterine corpus endometrial carcinoma (UCEC), Uterine carcinosarcoma (UCS), lung squamous carcinoma (LUSC), rectal adenocarcinoma (READ), and colon adenocarcinoma (COAD).

GPR15 showed a low mutation rate compared with hotpots oncogenes among all TCGA cohorts (Figure S2). It is most frequently mutated in uterine corpus endometrial carcinoma (UCEC), uterine carcinosarcoma (UCS), lung squamous carcinoma (LUSC), rectal adenocarcinoma (READ), and colon adenocarcinoma (COAD) (Figure 1A). We performed somatic mutations analysis on these five cancers. The mutational distribution and protein domains for GPR15 with labelled hotspots are shown in Figure S3. Most mutations in GPR15 are missense mutations while the minority mutational pattern is heterogenous, and the variant classification varies from frameshift deletion (COAD), frameshift insertion (LUSC), and nonsense mutation (LUSC, READ) to missense mutation (Figure 2). Moreover, it is worth noting that GPR15 in COAD is both hypermutated and significantly downregulated compared to that in normal tissues. This pattern implies that alterations in GPR15-meditor T-cell homing [8] may have undiscovered effects on the pathophysiology of COAD.

### 2.2. Integrated Network Analysis of GPR15

**Figure 3:**
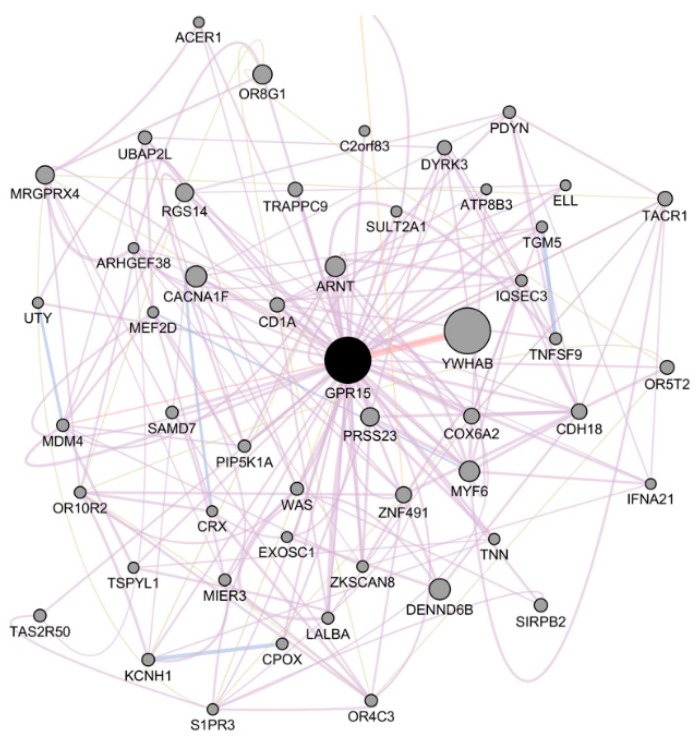
The integrated network of *GPR15***.** The edge color represents supporting data. Pink means the line is based on physical interactions. Purple means the line is generated from co-expression profiles. Blue means co-localization, and yellow stands for predicted interaction. Node size stands for its weight in the network.

To obtain more functional insights for *GPR15*, we performed integrative network analysis on GPR15 [38] as shown in Figure 3. The network was built upon co-expression, physical interaction, genetic interaction, shared protein domains, and pathway data, where we found that the most related protein is YWHAB, and the linkage is supported by direct physical interactions. *YWHAB* encodes the protein 14-3-3 protein beta/alpha, which plays a role in mitogenic signaling and cell cycle machinery [39]. Integrated network analysis revealed that, apart from immunity control, GPR15 may have effects on cell growth, thereby affecting carcinogenesis. The top five GPR15-related genes with the highest scores are shown in Table 1. 

Pathway analysis was conducted on the top 50 genes in the network to illustrate their biological function by the Reactome platform. We found Butyrate Response Factor 1 (BRF1) binding and tristetraprolin (TTP, ZFP36) binding as the two most significant pathways. Both pathways involve *YWHAB,* which further implies the close interaction between GPR15 and *YWHAB*. The top five most related pathways are shown in Table 2 and Table S1.

### 2.3. Pan-Cancer Analysis of GPR15 Expression and Prognostic Association

To evaluate *GPR15* expression and prognosis in a pan-cancer manner, we used the pre-train multiple variate Cox regression model, which combined specific gene expression value and basic clinical data provided by OncoLnc [46] to identify the TCGA cohorts of which the prognosis is significant with the GPR15 expression value. We found that the prognoses of the four cancer types, COAD, HNSC, LUAD, and stomach adenocarcinoma (STAD), are possibly (*p* < 0.15) associated with GPR15 expression (Table 3, Table S2). In addition, based on Cox coefficients, the hazards of COAD, HNSC, and LUAD were found to be negatively associated with GPR15 expression, whereas the expression of GPR15 was positively correlated with the hazard of STAD. 

**Figure 4:**
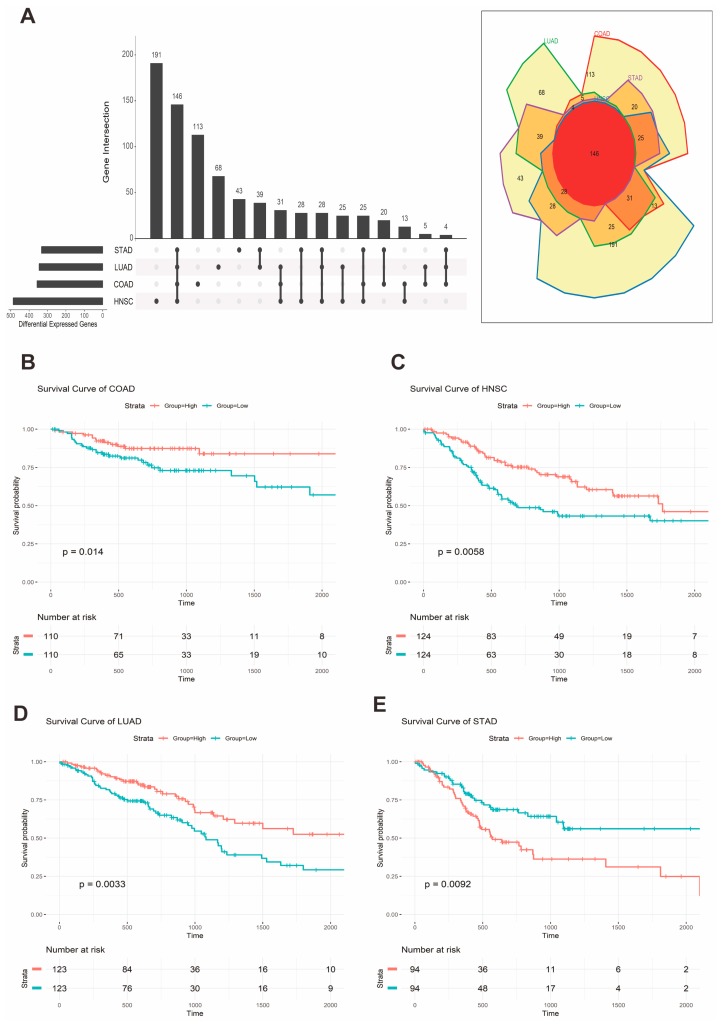
Differential gene expression (DEG) analysis results and Kaplan Meier (KM) plots. (**A**)**.** Upset and Venn diagram of DEGs in COAD, HNSC, LUAD, and STAD. (**B**–**E**). KM plots of the GPR15 expression groups in COAD, HNSC, LUAD, and STAD.

Then, we stratified the patients in each cohort based on the expression median into high- and low-expression groups. Afterward, we built Kaplan Meier (KM) plots for the GPR15 group in COAD, HNSC, LUAD, and STAD separately. We found that the prognoses of COAD (*p* = 0.014), HNSC (*p* = 0.0058), LUAD *(p* = 0.0033), and STAD (*p* = 0.0092) were significantly correlated with the GPR15 expression groups (Figure 4B–E).

GPR15 can reduce the inflammation level in the large intestine by controlling T-cell homing [8]. We thus hypothesized that the high expression of GPR15 in COAD can contribute to the homing and infiltration of FOXP3+ regulatory T cells (T_regs_), which in turn boost the immunity response of the tumor and results in a better prognosis. This pattern was also observed in HNSC and LUAD, which suggests that GPR15 may perform similar immunity control functions in head, neck, and lung tissues. However, the observed upregulation of GPR15 in STAD implies poorer prognosis, which may suggest the opposite effects of GPR15 on stomach tissue.

### 2.4. Commonly Upregulated Gene Set in High GPR15 Groups of COAD, HNSC, LUAD, and STAD

To dissect the effects of the expression of *GPR15* in a genome-wide manner, we performed differential gene expression (DEG) analysis [47,48] on the *GPR15* low-expression group compared to the GPR15 high-expression group in the four cancer types. We found that 357, 487, 346, and 333 genes were differentially expressed in COAD, HNSC, LUAD, and STAD, respectively (Supplementary Material). The profiles of the top 200 differential expressed genes in COAD, HNSC, LUAD, and STAD are shown in Figure S4. Interestingly, we found that 146 genes were commonly upregulated (Figure 4A). These genes are defined as a commonly upregulated gene set (CUPGS). This considerable number of CUPGS implies a shared regulatory mechanism of *GPR15* in COAD, HNSC, LUAD, and STAD. 

**Figure 5:**
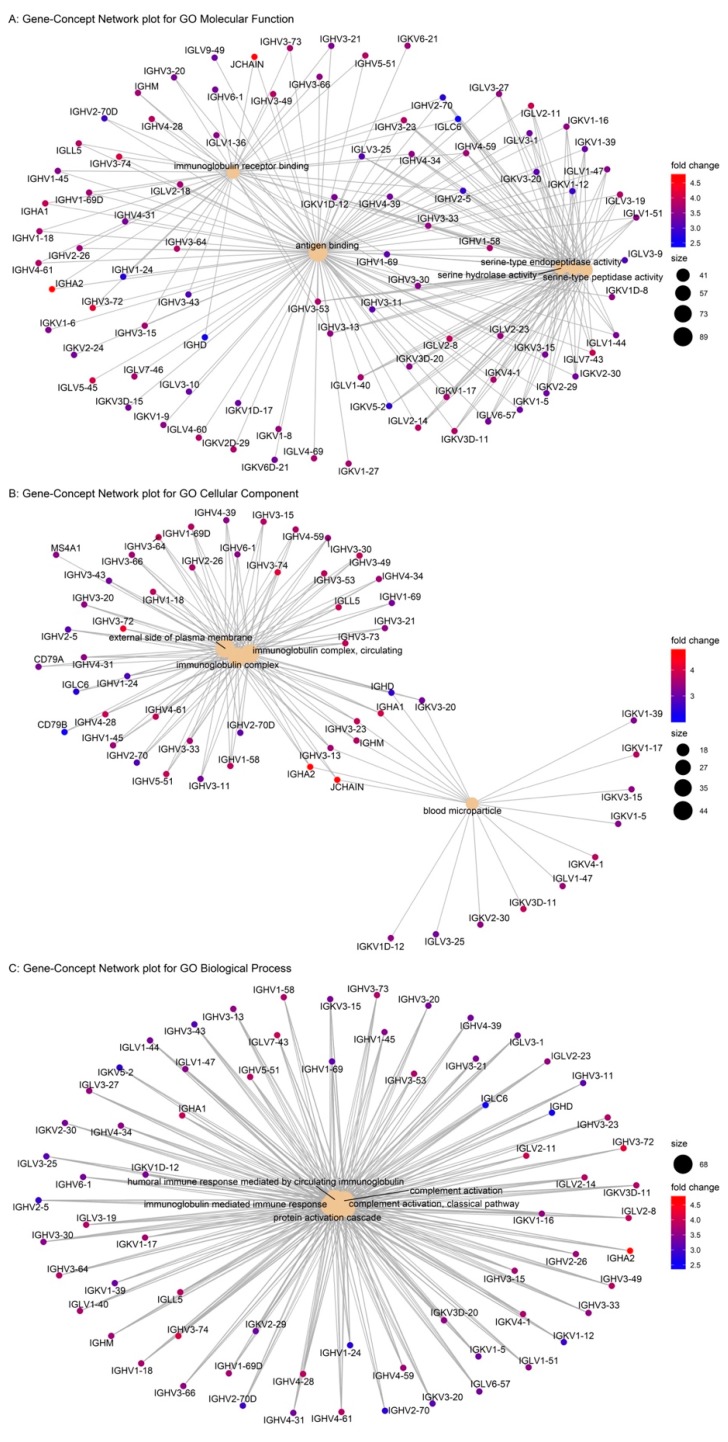
Gene-concept network for commonly upregulated gene set (CUPGS). The size of the GO term stands for the number of genes in the CUPGS that is annotated based on the term. Color scale of the gene name stands for the mean fold-change in the high GPR15 expression groups compared to the low GPR15 expression groups among COAD, HNSC, LUAD, and STAD.

GO enrichment analysis of the CUPGS was conducted and the results are shown in Figure S5A–C. Intuitively, we found that these genes were significantly enriched in the functional category of antigen binding (*p* = 2.41 × 10^−159^), cellular component of immunoglobulin complex (*p* = 1.32 × 10^−50^), and the biological process of various immunological response processes. The results of KEGG pathway analysis are shown in Figure S5D–F, which also showed that the CUPGS enriched the categories of the B-cell receptor immunology pathway, intestinal immune network for immunoglobulin A (IgA) production, and primary immunology. The GO enrichment results are listed in Table 4. The gene-concept network for CUPGS is depicted in Figure 5. Surprisingly, besides the established function of T-cell homing of *GPR15*, these CUPGS were also significantly associated with B-cell meditated immunity, and these genes were upregulated in the high *GPR15* expression groups, which suggests that *GPR15* exerts a broader immunological impact.

### 2.5. Association between GPR15 Expression Levels and the Immune Cell-Infiltrating Levels in Cancer

**Figure 6:**
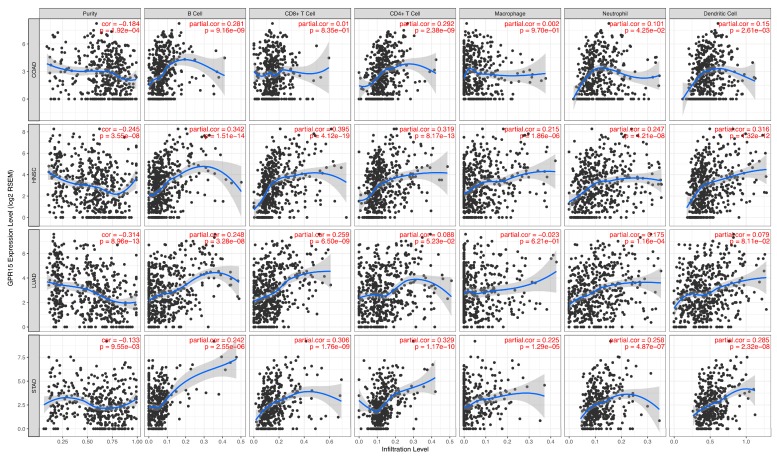
Correlations between expression level of GPR15 and immune cell infiltration levels in COAD, HNSC, LUAD, and STAD generated from TIMER.

Together, the results of the enrichment analysis revealed that the regulatory role of GPR15 in the four cancers is strongly correlated to immunity function. To support these findings, we investigated the association between GPR15 expression levels and immune cell infiltration levels in the tumor microenvironment using TIMER (Figure 6, Table S3). We found that the GPR15 expression value was significantly negatively correlated with tumor purity in all four types of cancer. As for T cells, for three types of cancer, excluding COAD, the GPR15 expression value was significantly positively correlated with CD8+ T cell infiltration. In COAD, HNSC, and STAD, the GPR15 expression value was significantly positively correlated with CD4+ T cell infiltration. Even with similar immune profiles, the prognosis of STAD is the opposite from the other three types of cancer, which implies different underlying mechanisms.

### 2.6. 3D Structure Modeling of GPR15 

**Figure 7:**
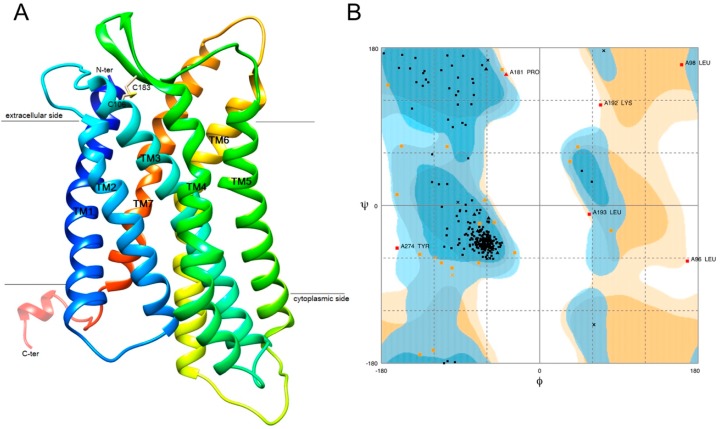
Homology modeling result of GPR15. (**A**) Predicted seven transmembrane structure (7TM) of GPR15. Different colors indicate various domains. In addition, there is a disulfide bond between Cys106 and Cys183. (**B**) The Ramachandran plot result of GPR15.

Based on all the aforementioned analyses, we hypothesized that GPR15 could be a novel target of cancer immunotherapy. Recent studies have shown that the known natural ligands of GPR15 are all agonists [21,22]. We thus put more of an emphasis on drug discovery specifically for STAD to identify potential inhibitors of GPR15. There is no crystal structure available for GPR15. Thus, we performed homology modeling for the 3D structure of human GPR15. Template-based modeling is the most common approach to explore the relationships between the three-dimensional coordinates of unknown proteins and their homologs. The GPR15 sequence was searched against the PDB-BLAST for similar template selection, and type-1 angiotensin II receptor (PDB:4YAY) was selected, with a sequence identity of 32.64% and query coverage of 30 to 317 aa (Figure S6). A total of 10 models were generated and further validated by the SAVE server. The best predicted model structures were further refined by calculation of the probability density function (pdf) and discrete optimized potential energy (DOPE). The 3D model had a DOPE score of –15,495.15, which was the lowest against the predicted other models. Also, the Ramachandran plot showed 90.9% of the residues in the allowed region that depicted the stability of the predicted model. The results of the homology modelling of GPR15 are shown in Figure 7. 

### 2.7. Structure–Function Relationship-Based Binding Site Prediction 

**Figure 8:**
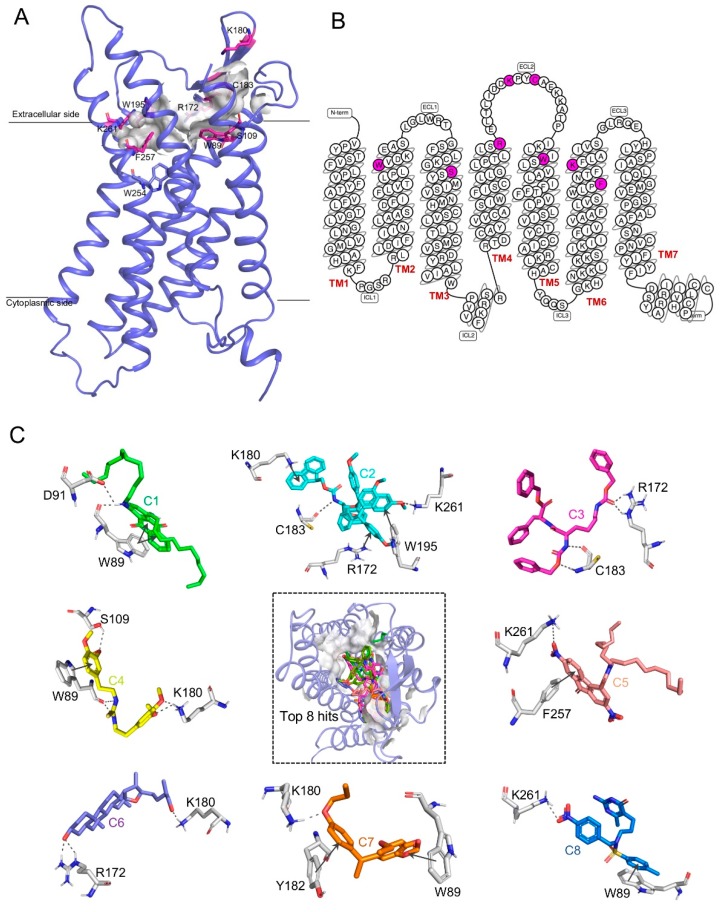
Ligand binding landscape of GPR15 (**A**) The schematic graph of the predicted binding site of GPR15. The predicted binding site in our 3D structure is a traditional orthosteric binding site in the vicinity of the highly conserved residue (TRP254, W^6.48^) of family A GPCRs [50]. The key residues are shown in purple-red sticks. (**B**) The snake diagram, generated via GPCRdb [51], of the predicted active side residues (purple) interacting with the ligands. (**C**) The predicted binding mode between GPR15 and ligands at the active site pocket (dashed box). The protein–ligand interactions of representative docking poses of the top eight hits are displayed around. Different ligands are represented by different colored sticks. Hydrogen bonds are illustrated by purple lines, and Pi–pi and Pi–cation interactions are marked by a black line.

The structure–function relationship of GPR15 is helpful in drug design. We identified its structure–function relationship using the Cofactor server [49]. We found that TRP89, SER109, ARG172, LYS180, CYS183, TRP195, PHE257, and LYS261 residues were located in the active region in GPR15. Active site regions were largely located in the extracellular regions of seven transmembrane domains, where the potential leads can bind and play a crucial role in signal transduction. A schematic representation of the ligand binding site is shown in Figure 8A,B. Also, cross-validation of the predicted residues at the active region was further supported by the results produced in the Site Finder tool of the MOE suite. Amino acid residues within 5 Å of the active were used for the generation of the receptor grid of GPR15 that was used for virtual screening. 

### 2.8. Virtual Screening and Molecular Docking Results

We utilized the virtual screening technique to identify potential antagonists exhibiting an adequate binding affinity. We started with a chemical database consisting of 62,500 small molecules and isolated a set of compounds satisfying the threshold of a high docking score. After the first round of filtration, we obtained 733 compounds via shape-based virtual screening. This shape-based screening approach utilizes the concept of the shape of binding pockets and electrostatic potential resemblance to select new molecules, which may show similar binding modes to the binding pocket. These 733 molecules were subjected to re-docking. Docking of the selected ligands was achieved to obtain the top conformations of the selected 733 molecules into the predicted GPR15 binding site. Finally, based on the docking scores, with the threshold values fixed between −13.00 and −8.00, only the top eight screened compounds ranked by the lowest binding energy were identified as potential antagonists for GPR15. The interactions analysis for the eight hits is given in Table 5 and Figure S7, and their 2D structures are given in Figure S8.

### 2.9. MD Simulations and Binding Free Energy Analysis 

**Figure 9:**
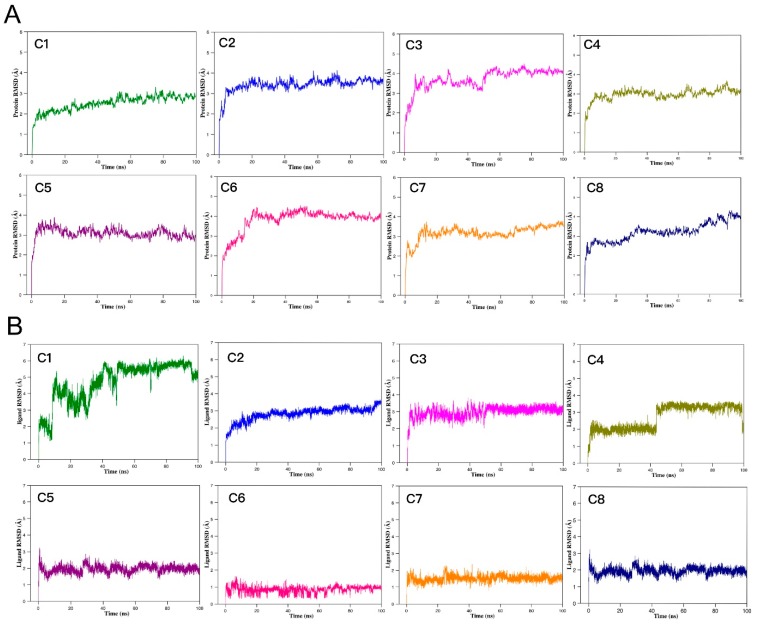
Root-mean-square deviation (RMSD) results of the GPR15–ligand complex systems as a function of time during the MD simulations. (**A**) RMSDs of backbone atoms for GPR15–ligand complexes. All the complexes are stable and attain the stability soon after reaching 20 ns. (**B**) RMSDs of heavy atoms for screened ligands. Compound 5, 6, 7, and 8 showed lower fluctuations among the eight hits in RMSD.

We performed MD simulation of the top eight potential complexes to measure the stability of the protein–ligand complex. RMSD (root-mean-square deviation) profiles of the protein are shown in Figure 9A, which indicates that all systems were stable during the entire simulation run and could be used for further analysis. The RMSD of ligand-heavy atoms was also conducted to predict the stability of the atoms in docked complexes (Figure 9B). Compounds 5−8 exhibited a consistently lower RMSD (<2.1 Å), suggesting that that these compounds formed stable complexes with GPR15. We selected four hits with lower ligand RMSD values for further interaction analysis and explored the ligand binding mode in the protein based on the occupancy of hydrogen throughout the simulation time. Compound 5 showed more than 90% salt bridge interaction with Lys261 (Figure S9) in the MD trajectories. The fluctuation in RMSD was further supported by the MM/PBSA results (Table S4), which showed that compound 5 (C_34_H_47_O_6_N_3_) had a stronger binding affinity (lowest binding free energy) among the hits with consistently lower RMSD values. Combining all the structural analyses, we identified compound 5 as a promising candidate for GPR15 inhibition. 

## 3. Discussion and Conclusions

GPCRs are well-established crucial participants in various signal transduction pathways and are major targets in drug design. Until now, more than 134 GPCRs as targets for drugs have been approved in the United States or European Union [52]. Although the endogenous ligand is not known, O-GPCRs are still popular targets with specificity in many therapeutic approaches. There is a broad range of indications linked to orphan GPCRs, including cancers, thus O-GPCRs may be utilized as clinical therapeutic targets in cancer therapy [53].

In this study, we demonstrated a novel integrative pan-cancer analysis workflow and conducted a comprehensive analysis from upstream omics to downstream drug discovery of GPR15 in cancer. Our study reported GPR15 expression and mutation levels across all cancers; the correlation between its expression and cancer prognosis; an investigation of genes with similar GPR15 expression patterns in COAD, HNSC, LUAD, and STAD; and 3D structure modeling of GPR15 to virtually screen its antagonists.

Our study provided evidence of the associations between GPR15 expression and cancer immunity. We analyzed CUPGS in COAD, HNSC, LUAD, and STAD to investigate the functions of co-expression genes with similar GPR15 expression patterns. Nearly all CUPGS were enriched in the immune-related function. GPR15 was proven to mediate regulatory T cells (T_regs_) to migrate to the large intestine and reduce inflammation in the mouse model [8], but it is preferentially expressed on human effector T cells [13]. Further research supported that GPR15-dependent human CD8^+^ T cells can migrate into the inflamed gut, and GPR15 can also help dendritic epidermal T cells migrate to the skin [15]. Mounting evidence suggests that GPR15 may play a role in the pathological process of chronic inflammatory diseases [54]. In normal tissue, chronic inflammation is a well-acknowledged risk factor for cancers. It could gradually generate an immunosuppressive tumor microenvironment, which allows mutated cells to evade the surveillance of the immune system, causing cancer eventually [17]. However, in malignant cancer tissue, immune infiltration, which is often depicted as “inflammation”, suggests better prognosis [55]. We found that GPR15 expression is significantly positively associated with the prognoses of COAD, HNSC, and LUAD, and significantly negatively associated with STAD. Based on clinical and transcriptomic analyses, we can hypothesize that GPR15 could influence cancer prognosis through downstream immunological effectors. Moreover, the tumor microenvironment dissection via TIMER also supports possible CD8+ T cell and CD4+ T cell infiltration mediated through GPR15.

A recent study has shown that GPR15 was deorphanized and its known natural ligands are all agonists [21,22]. From another point of view, designing inhibitors for GPR15 could provide some clues for the treatment of STAD and help the functional study of GPR15 at the molecular level for experimental biologists. Therefore, we performed virtual screening for GPR15 antagonists and predicted the protein–ligand interaction of the top eight compounds. MD simulation and free energy calculation conducted on the top eight compounds led to the discovery of the best compound, compund 5 (C_34_H_47_O_6_N_3_), which could be a hit for novel drugs targeting STAD.

Together, our analysis functionally annotated GPR15 expression in a pan-cancer manner and identified potential inhibitory agents that target GPR15. Our study provided evidence of the associations between GPR15 expression and cancer immunity. Our results provide new clues regarding GPR15′s role in carcinogenesis and new insights into cancer therapy targets. Also, this novel comprehensive omics-based workflow could be utilized for the hypothesis generation of new targets in cancer.

## 4. Methods

### 4.1. Pan-Cancer Mutational Data Retrieval

We retrieved the mutation annotation format (MAF) files of the Cancer Genome Atlas (TCGA) [27] cohorts using the R package “TCGAbiolinks” [56] on 15 October 2018. The TCGA program provided us with multiple versions of somatic mutation data sets, which were generated using different workflows, and we selected the data set “MuTect2 Variant Aggregation and Masking” [57] because it encompassed more mutations than the others. Besides, according to a comparison study of mutation callers [58], MuTect2 has the highest recall and robustness. A total number of 33 cancer types and 9914 cancer samples were included in this study. Mutational summary and landscape plots were performed by R package “maftools” [59]. The mutant rate plot of GPR15 was available from the National Cancer Institute GDC Data Portal (https://gdc.cancer.gov).

### 4.2. Pan-Cancer GPR15 Expression Profile Analysis

We used GEPIA [60] to interactively analyze the expression profile of *GPR15* among 33 cancer types, 9736 tumors, and 8587 paired normal samples from the TCGA and the Genotype-Tissue Expression (GTEx) project [61]. We used the limma [48] backend with the threshold of log2 fold-change >1 and *q*-value <0.05 to detect cancer types exhibiting differential expressed *GPR15* compared to matched normal samples. Differential expression of GPR15 was validated from the UALCAN database (http://ualcan.path.uab.edu/index.html). The Spearman’s correlation between the GPR15 expression value and immune infiltration level in 4 types of human cancer (COAD, HNSC, LUAD, and STAD) was calculated via TIMER (Tumor IMmune Estimation Resource) [62], and visualized by its “Gene” module. 

### 4.3. Integrated Network Analysis of GPR15

To obtain more functional insights into *GPR15*, we used GeneMANIA [38] and Cytoscape [63] to perform integrated network analysis. The integrated network consists of co-expression data from Gene Expression Omnibus [64] (GEO), physical and genetic interaction data from BioGRID [40], predicted protein interaction data based on orthologue using the Interologous Interaction Database [65] (I2D), and pathway molecular interaction data from BioGRID. In an algorithmic perspective, the integrated network analysis can be divided into two parts. Firstly, we used a linear regression-based algorithm to calculate a single composite functional association network from multiple network data sources (co-expression, physical interaction, genetic interaction, shared protein domains, pathway data, and so on). Second, a variation of the Gaussian field label propagation algorithm was utilized to assign a score (the discriminant value) to each node in the network. This score reflects the computed strength of association between gene pairs [66]. We also used the Reactome [67] platform to conduct pathway analysis of the predicted genes.

### 4.4. Survival Analysis of GPR15 Expression

We used OncoLnc [46] to determine the cancer type o which the prognosis is potentially associated with *GPR15* expression. Then, we used the R package “TCGAbiolinks” to retrieve the corresponding clinical and expression data. We stratified patients in each associated cohort into “high” and “low” groups based on the median expression value of *GPR15*. The Kaplan–Meier method was utilized to estimate the survival function, and we used the log-rank test to evaluate the significance between two groups. Survival analysis and corresponding visualization were performed using the R package “survival” and “survminer” [68], respectively.

### 4.5. Gene Differential Expression Analysis

We used standalone limma and voom [47] pipeline to identify differentially expressed genes associated with *GRP15* expression, comparing tumor samples with high expression of *GPR15* to low-expression ones and applied the threshold of log_2_ fold-change >1 and adjusted *p*-value <0.01 to select for biological significant genes.

### 4.6. Commonly Upregulated Gene Set Identification and Annotation

We extracted differential expressed genes (DEGs) that were upregulated and shared among COAD, HNSC, LUAD, and STAD, and visualized these using the R package “UpsetR” [69] and “vennerable” [70]. These genes were collectively defined as the commonly upregulated gene set (CUPGS). We used the R package “clusterProfiler” [71] to perform gene ontology [72] and Kyoto Encyclopedia of Genes and Genomes (KEGG) [73] annotation of the CUPGS. 

### 4.7. 3D Structure Prediction and Validation of GPR15

We used BLASTP [74], which is implemented in NCBI (https://www.ncbi.nlm.nih.gov/), to search and align the best templates for 3D structural modeling of GPR15. We used BLAST-p to align GPR15 with similar PDB structures and protein sequences retrieved from the UniProt database (https://www.uniprot.org/). A template was identified from the NCBI-BLASTp program. Homology modeling was performed using the MODELLER program [75], where 10 models were built through the aligned templates, and Python scripts were executed for loop modeling and model refinement. Model selection was based on the parameters of the optimized loop, side-chain conformations, DOPE, Q-mean, Z-score, and maximum deviation. Structure refinement of the modeled GPR15 was performed using the KoBaMIN [76], a web server, in order to obtain the best conformation of the modeled structures resulting from MODELLER. Validation of the predicted model was performed using the Ramachandran plot generated by the Structure Analysis and Verification Server (SAVES) server (https://servicesn.mbi.ucla.edu/SAVES/). 

### 4.8. Active Site Prediction

The structure–function relationship for the predicted GPR15 model was established by exploring the active-site residues using the Cofactor server [49]. This approach was used to identify the biochemical function of the predicted GPR15 model and the potential binding region for its antagonists. The cross-validation of the predicted binding site was also conducted by the Site Finder tool of the MOE software. 

### 4.9. Screening of Potential Compounds Targeting GPR15

We screened the chemical library using the best homology model of GPR15 as the receptor structure. We performed shape-based virtual screening in the first round using the Surflex-Dock [77] module of the SYBYL software. The receptor was optimized and prepared for virtual screening with hydrogen atoms and charges added. A library of 62,500 compounds obtained from the Maybridge Library (55,975) and in-house compound library (6525) were used for the screening of compounds. The dataset compounds were converted to 3D coordinates and then minimized via the Powell method using 1000 iterations with a Tripose force field. We detected the surface in the predicted active sites and mapped an idealized active site ligand (called a protomol). Then, we applied five maximum conformations per fragment and five maximum poses per ligand with a 0.05 Å minimum (root-mean-square deviation) RMSD to dock in the defined Protomol region. The search area was set to 5 Å in the grid. We restricted the cutoff value (total score < –6) in the docking scoring function to eliminate false positive results. 

### 4.10. Molecular Docking

Docking experiments were performed via Surflex-Dock. Top hits were selected for docking and the receptor grid box was confined around the 5 Å area of the predicted active site radii. The Lamarckian genetic algorithm, a well-known docking algorithm, was used to conduct docking by setting the default parameters with 150 initial populations with randomly placed individuals and the maximum number of generations set to 27,000. A shortlist based on the consensus scoring function (Chem score + G Score + D Score + PMF Score) was generated. We then applied a cutoff (total score <–8) for the docking score function to eliminate false positive results. The lowest free binding energy was set as the criterion for the selection of the top poses.

### 4.11. Molecular Dynamics (MD) Simulations

We selected the top eight hits based on stability and protein–ligand interaction analyses. The initial structures for MD simulations originated from the representative docking pose form virtual screening. The GROMACS V5.1.3 package [78] was used to perform biophysical simulation of the eight complexes for 100,000 ps (100 ns), respectively. Membrane systems were constructed using the CHARMM-GUI Membrane Builder [79]. Proteins and lipids were presented using the CHARMM36 force field [80], and ligands were assigned to the CHARMM CGenFF [81]. All the systems were solvated in a cubic water box with a distance of 10 Å between the proteins, and the TIP3P model [82] was used for water molecules. Counter ions (0.15 M NaCl) were used to keep each system electrically neutral followed by a steepest descent energy minimization (~5000 steps). Subsequently, the minimized system was equilibrated into the NVT and NPT phases for 500 ps, and all bond lengths were restrained by using the LINCS method [83] with time steps of 2 fs. The temperature was set to 310 K and the pressure was maintained at 1.01325 × 10^5^ Pa (1 air pressure) using the Langevin piston method [84], which were controlled by a Nose-Hoover thermostat and Parrinello-Rahman barostat [85], respectively. The particle mesh Ewald algorithm (PME) [86] was utilized to compel long-range electrostatic interactions, and a 1.4-nm cut off for short-range van der Waals interactions was utilized. Sampling of the MD trajectories was carried out every 2.0 ps. Finally, 100 ns MD simulations for each system were performed for further analysis. Detailed simulations conditions are listed in Table S5.

### 4.12. MD Trajectories Analysis

The time course of the root-mean-square deviation (RMSD) from the respective initial structures was used to assess the stability of the proteins and ligand in different simulations. Hydrogen bonds were defined as hydrogen–acceptor at a distance less than 3.5 Å and donor–hydrogen–acceptor angle as more than 135°. Salt bridges were defined by oppositely charged atoms that were within 5 Å. All of the analyses were performed using the analysis tools implemented in GROMACS. In total, 100-ns trajectories (10,000 structure) of each MD system were analyzed after eliminating the rotational and translational movements. The trajectory images were visualized and analyzed with PyMol (https://pymol.org/2/) and VMD (http://www.ks.uiuc.edu/Research/vmd/).

### 4.13. Binding Free Energy Calculations

To estimate the corresponding relative binding affinities, the binding free energy for selected complexes was calculated using the molecular mechanics Poisson–Boltzmann surface area (MM-PBSA) method as implemented in the g_mmpbsa tool [87], which integrates functions from GROMACS and APBS [87]. For a protein–ligand complex, the lower the binding free energy, the higher the binding affinity. The calculation was based on the following equation:

(1) $$\begin{array}{l} \Delta G_{bind}=G_{complex}-G_{protein}-G_{ligand}\\ \Delta G_{bind}=\Delta E_{MM}+\Delta G_{PB}+\Delta G_{nonpolar}-T\Delta S\\ \Delta G_{bind}=G_{ele}+G_{vdw}+G_{SA}+G_{PA} \end{array},$$

where *∆E_MM_* is the sum of the van der Waals and electrostatic energy, *∆G_PA_* is the polar solvation energy, and ∆*G_SA_* is the non-polar solvation energy. The final, binding energy, *∆G_bind_*, was a relative value rather than an absolute value because the vibrational entropy contribution (*T∆S*) was not included in our calculation. In total, 100 snapshots at an interval of 10 ps from the last 10-ns trajectories during the stable phase were extracted as sampling for the calculations. 

## Figures and Tables

**Table 1:** Top five GPR15-related genes with highest scores.

Gene	Score	Network Group	Network Resource
*YWHAB*	0.22097643	Physical Interactions	BioGRID-small-scale-studies [40]
*YWHAB*	0.14404532	Physical Interactions	IREF-INTACT [41]
*TACR1*	0.038140558	Co-expression	Tateno-Hirabayashi 2013 [42]
*TAS2R9*	0.034424774	Co-expression	Hannenhalli-Cappola 2006 [43]
*SPDYE4*	0.031733938	Co-expression	Coelho-Hearing 2015 [44]
*GPR182*	0.029303862	Co-expression	Scholtysik-Kuppers 2015 [45]

**Table 2:** Top five pathways of genes in the integrated network.

Pathway ID	Pathway Name	*p*-Value	Entities Found
R-HSA-450385	Butyrate Response Factor 1 (BRF1) binds and destabilizes mRNA	0.00231	YWHAB;EXOSC1
R-HSA-450513	Tristetraprolin (TTP, ZFP36) binds and destabilizes mRNA	0.00231	YWHAB;EXOSC1
R-HSA-525793	Myogenesis	0.00636	MYF6;MEF2D
R-HSA-375170	CDO in myogenesis	0.00636	MYF6;MEF2D
R-HSA-388396	GPCR downstream signaling	0.00967	GPR15

**Table 3:** Top 10 potential cancer types whose prognosis is associated with GPR15.

Cancer	Cox Coefficient	*p*-Value	Rank
STAD	0.27	0.002	269
HNSC	−0.205	0.006	707
LUAD	−0.161	0.039	3711
COAD	−0.159	0.150	4299
READ	−0.328	0.160	2696
LUSC	0.07	0.330	6956
KIRC	−0.059	0.480	13,174
LAML	0.078	0.510	9516
ESCA	0.021	0.880	14,833

“Rank” stands for the expression abundance rank among all genes.

**Table 4:** Top 10 enriched GO terms for each GO category. BP stands for biological process, CC stands for cellular component, MF stands for molecular function.

ID	Description	*p*-Adjust	Category
GO:0006958	complement activation, classical pathway	2.78 × 10^−120^	BP
GO:0002455	humoral immune response mediated by circulating immunoglobulin	3.03 × 10^−117^	BP
GO:0006956	complement activation	2.21× 10^−112^	BP
GO:0072376	protein activation cascade	1.16 × 10^−107^	BP
GO:0016064	immunoglobulin mediated immune response	1.36 × 10^−104^	BP
GO:0019724	B cell mediated immunity	1.66 × 10^−104^	BP
GO:0002429	immune response-activating cell surface receptor signaling pathway	3.22 × 10^−91^	BP
GO:0006959	humoral immune response	4.60 × 10^−91^	BP
GO:0002768	immune response-regulating cell surface receptor signaling pathway	8.99 × 10^−94^	BP
GO:0002460	adaptive immune response based on somatic recombination of immune receptors	1.21 × 10^−91^	BP
GO:0019814	immunoglobulin complex	1.32 × 10^−80^	CC
GO:0042571	immunoglobulin complex, circulating	3.13 × 10^−77^	CC
GO:0009897	external side of plasma membrane	1.50 × 10^−44^	CC
GO:0072562	blood microparticle	2.80 × 10^−18^	CC
GO:0098802	plasma membrane receptor complex	0.513620478	CC
GO:0042101	T cell receptor complex	0.513620478	CC
GO:0008180	COP9 signalosome	0.721923256	CC
GO:0043235	receptor complex	0.721923256	CC
GO:0000788	nuclear nucleosome	0.721923256	CC
GO:0005771	multivesicular body	0.754761177	MF
GO:0003823	antigen binding	2.41 × 10^−159^	MF
GO:0034987	immunoglobulin receptor binding	2.28 × 10^−71^	MF
GO:0004252	serine-type endopeptidase activity	2.71 × 10^−46^	MF
GO:0008236	serine-type peptidase activity	9.31 × 10^−45^	MF
GO:0017171	serine hydrolase activity	1.40 × 10^−44^	MF
GO:0005068	transmembrane receptor protein tyrosine kinase adaptor activity	0.03233891	MF
GO:0042834	peptidoglycan binding	0.055957242	MF
GO:0031210	phosphatidylcholine binding	0.100244352	MF
GO:0050997	quaternary ammonium group binding	0.100244352	MF
GO:0035591	signaling adaptor activity	0.102967022	MF

**Table 5:** The docking score and predicted protein–ligand interaction of the top eight compounds selected in virtual screening.

Compound No.	Molecular Formula	Weight (g/mol)	Docking Score	Noncovalent Interactions	Residues
C1	C_38_H_58_O_2_N_2_	576.91	−11.63	2 Pi–pi, 2 H–bond	TRP89, ASP91
C2	C_60_H_55_O_8_N_1_	918.09	−11.15	1 Pi–pi, 2 Pi–cation, 1 H–Bond	LYS180, ARG172, TRP195, LYS261
C3	C_38_H_41_O_7_N_3_	653.77	−10.79	2 H–Bond	CYS183, ARG172
C4	C_21_H_28_O_4_N_2_S	404.53	−10.28	1 Pi–pi, 2 H–bond	TRP89, SER109, LYS180
C5	C_34_H_47_O_6_N_3_	593.76	−10.11	1 Pi–pi, 1 Salt–bridge	PHE257, LYS261
C6	C_27_H_46_O_3_	418.66	−8.72	2 H–bond	ARG172, LYS180
C7	C_20_H_24_O_4_	328.41	−8.3	2 Pi–pi, 1 H–bond	TRP89, TYR182, LYS180
C8	C_22_H_27_O_5_N_5_S	473.55	−8.29	1 Salt–bridge,1 Pi–pi	LYS261, TRP89

## Supplementary Materials

Supplementary materials can be found at https://www.mdpi.com/1422-0067/20/24/6226/s1.

## Abbreviations

ACC	Adrenocortical carcinoma
BLCA	Bladder urothelial carcinoma
BRCA	Breast invasive carcinoma
CESC	Cervical squamous cell Carcinoma and endocervical adenocarcinoma
CHOL	Cholangiocarcinoma
COAD	Colon adenocarcinoma
DLBC	Diffuse large B-cell lymphoma
ESCA	Esophageal carcinoma
GBM	Glioblastoma multiforme
HNSC	Head and neck squamous cell carcinoma
KICH	Kidney chromophobe
KIRC	Kidney renal clear cell carcinoma
KIRP	Kidney renal papillary cell carcinoma
LAML	Acute myeloid leukemia
LGG	Lower grade glioma
LIHC	Liver hepatocellular carcinoma
LUAD	Lung adenocarcinoma
LUSC	Lung squamous cell carcinoma
MESO	Mesothelioma
OV	Ovarian serous cystadenocarcinoma
PADD	Pancreatic adenocarcinoma
PCPG	Pheochromocytoma and Paraganglioma
PRAD	prostate adenocarcinoma
READ	Rectum adenocarcinoma
SARC	Sarcoma
SKCM	Skin cutaneous melanoma
STAD	Stomach adenocarcinoma
TGCT	Testicular germ cell tumors
THCA	Thyroid carcinoma
THYM	Thymoma
UCEC	Uterine corpus endometrial carcinoma
UCS	Uterine carcinosarcoma
UVM	Uveal Melanoma
