# Supplementary material for: An Integrated Pan-Cancer Analysis and Structure-Based Virtual Screening of GPR15
Source: Int J Mol Sci. 2019 Dec 10;20(24):6226. doi: 10.3390/ijms20246226 (PMC6940937; doi:10.3390/ijms20246226)
Supplement: Supplementary file 1 [file ijms-20-06226-s001.pdf]

Supporting Information

An Integrated Pan-Cancer Analysis and Structure-Based Virtual Screening of GPR15

Yanjing Wang [1,†], Xiangeng Wang [1,†], Yi Xiong [1], Cheng-Dong Li [1], Qin Xu[1], Lu Shen[2], Aman Chandra Kaushik [4,*] and Dong-Qing Wei [1,3,*]

[1] State Key Laboratory of Microbial Metabolism, School of Life Sciences and Biotechnology, and Joint Laboratory of International Cooperation in Metabolic and Developmental Sciences, Ministry of Education, Shanghai Jiao Tong University, Shanghai 200240, China; wangyanjing@sjtu.edu.cn (Y.W.); wangxiangeng@sjtu.edu.cn (X.W.); xiongyi@sjtu.edu.cn (Y.X.); lcd805728463@sjtu.edu.cn (C.-D.L.) xuqin523@foxmail.com (Q.X.)

[2] Bio-X Institutes, Key Laboratory for the Genetics of Developmental and Neuropsychiatric Disorders, Ministry of Education, Shanghai Jiao Tong University, Shanghai 200030, China; yoyomailer@sjtu.edu.cn (L.S.)

[3] Peng Cheng Laboratory, Vanke Cloud City Phase I Building 8, Xili Street, Nanshan District, Shenzhen 518055, China

[4] Wuxi School of Medicine, Jiangnan University, Wuxi 214122, China

* Correspondence: dqwei@sjtu.edu.cn (D.-Q.W.); amanbioinfo@sjtu.edu.cn (A. C.K.)

† These authors contributed equally to this work.

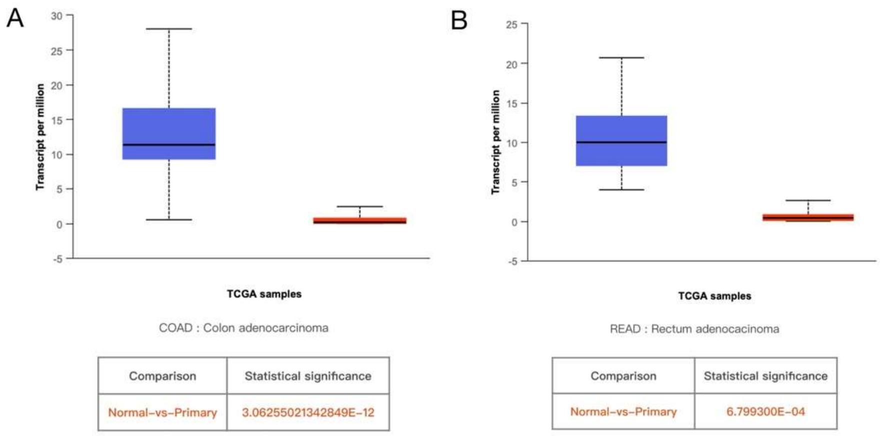

Figure 1. Differential of GPR15 between tumor and normal in COAD and READ.

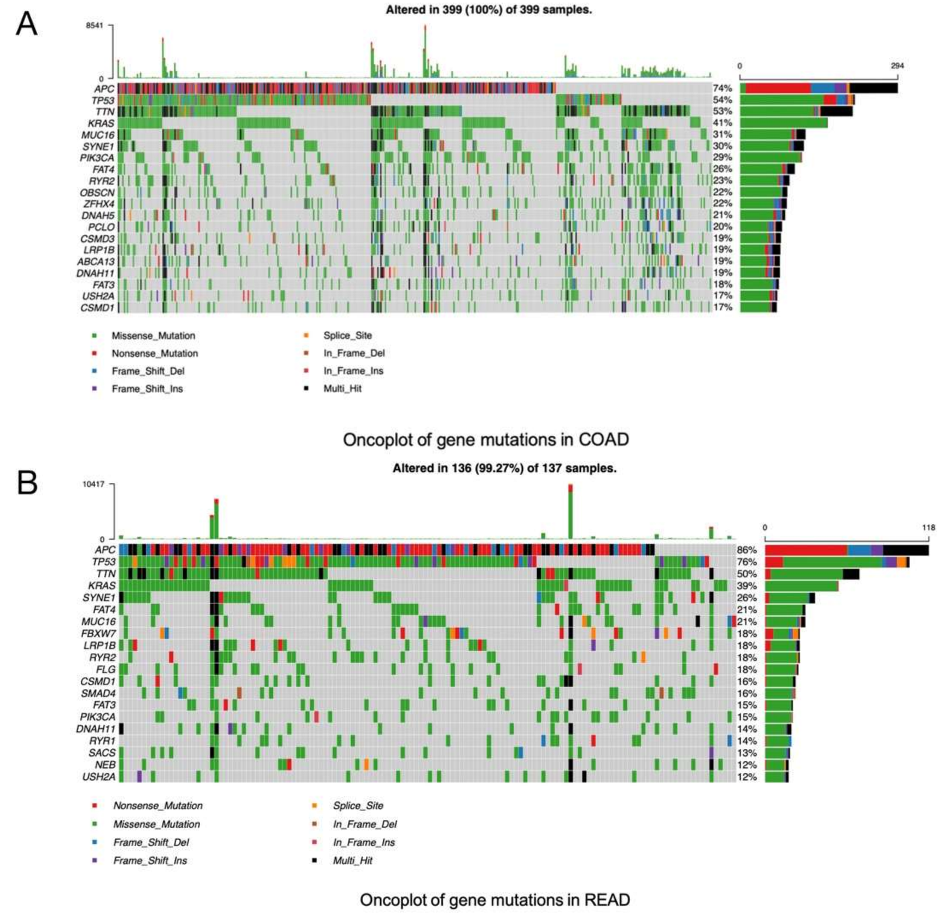

Figure 2. Mutational landscape of COAD and READ. A. Waterfall plot of the mutations among 399 COAD patients. B. Waterfall plot of the mutations among 137 READ patients. Top 20 frequently mutated genes are listed. .

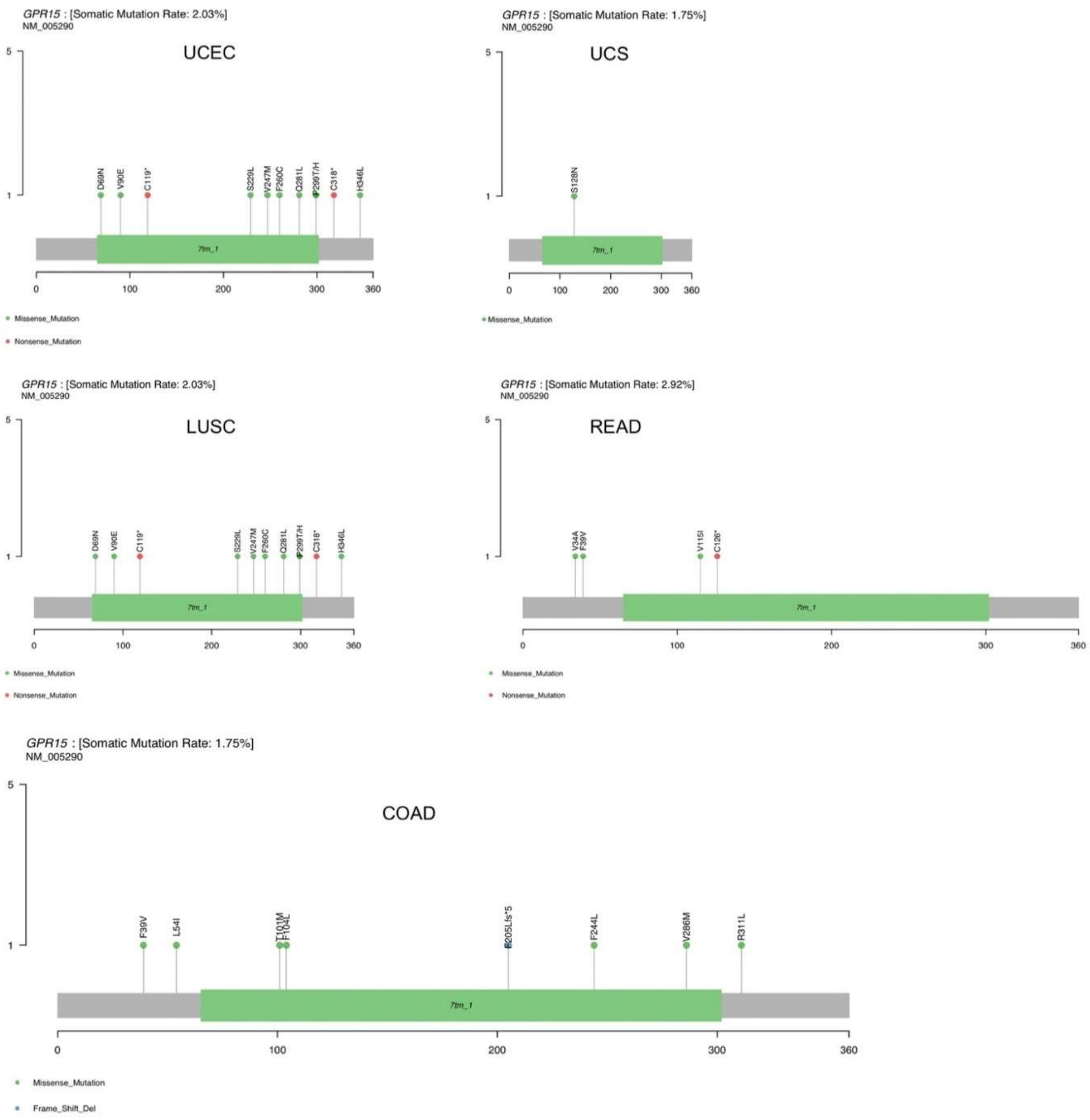

Figure 3. Mutational pattern of GPR15 in 5 cancers. Lollipop plot that depicts the mutational distribution and protein domains for GPR15 in five different cancers with labelled hotspots.

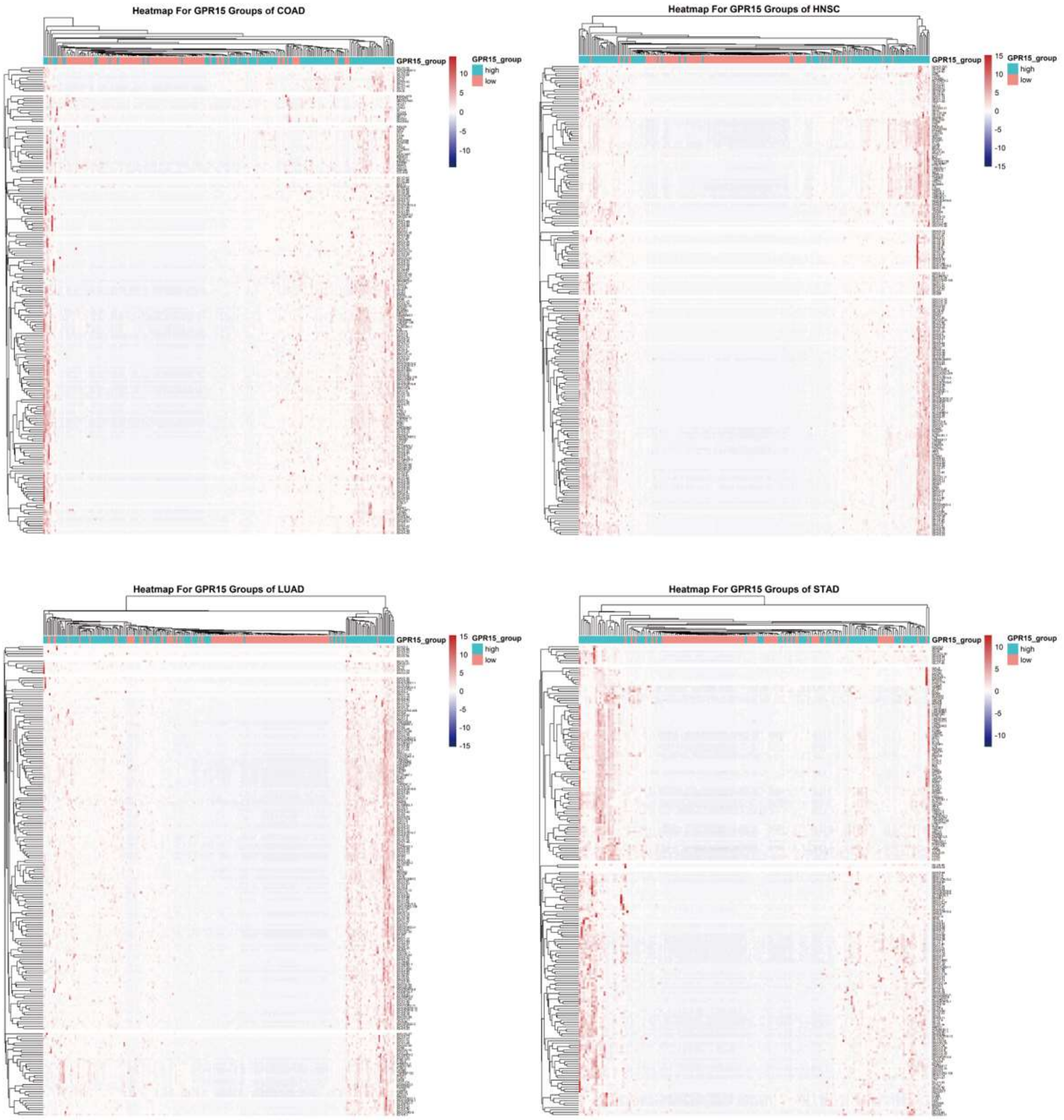

Figure 4. Heat maps for top 200 genes with altered expression between the GPR15 high expression and *GPR15* low expression groups.

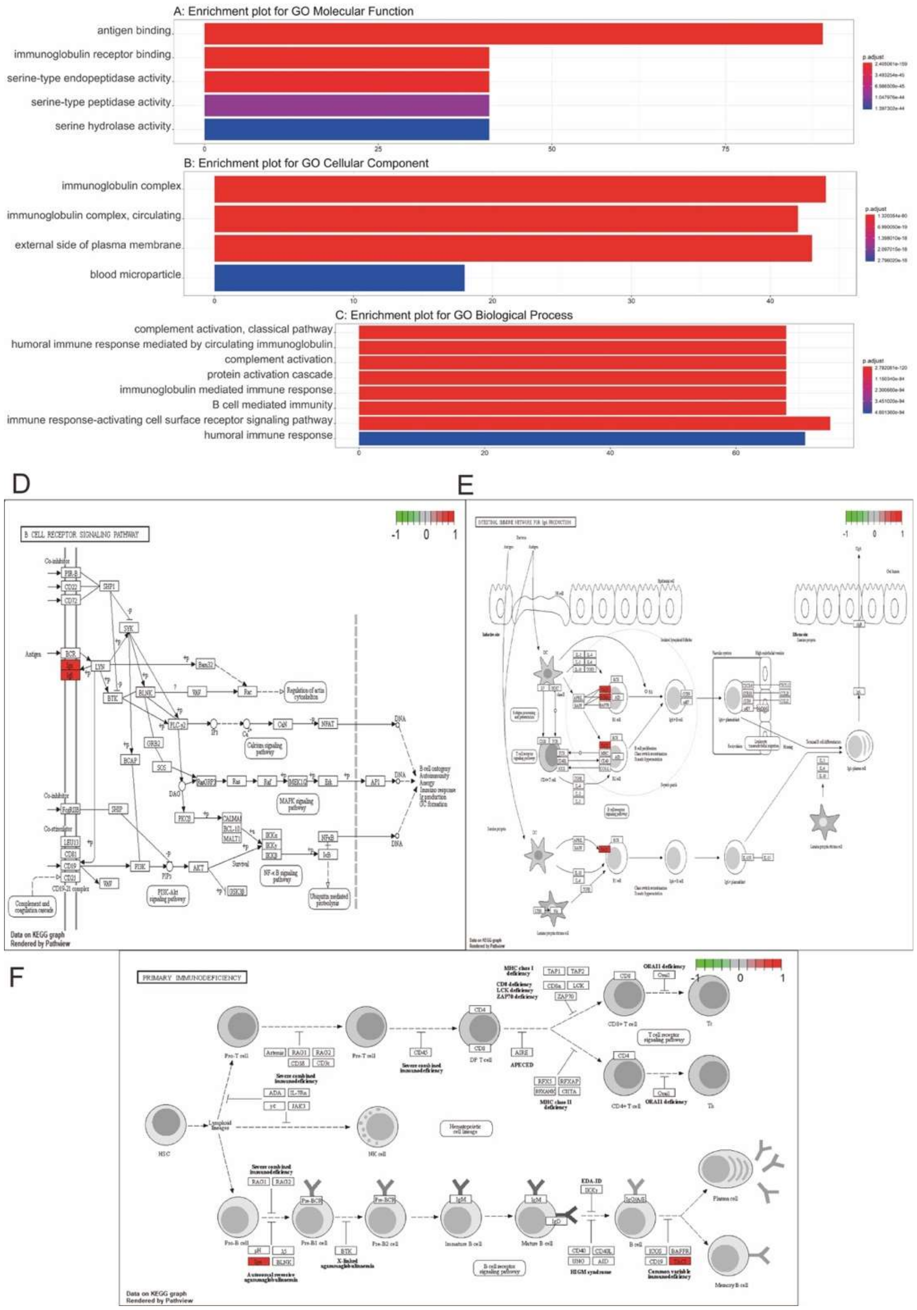

Figure 5. Enrichment results of CUPGS. A–C. GO enrichment results of molecular function, cellular component, and biological process. Color scale stands for the significance of each specific GO term. Bar length stands for the number of common altered genes within a specific GO term. D–F. KEGG enrichment results. Gene name in red means that the gene in a specific pathway is in the CUPGS.

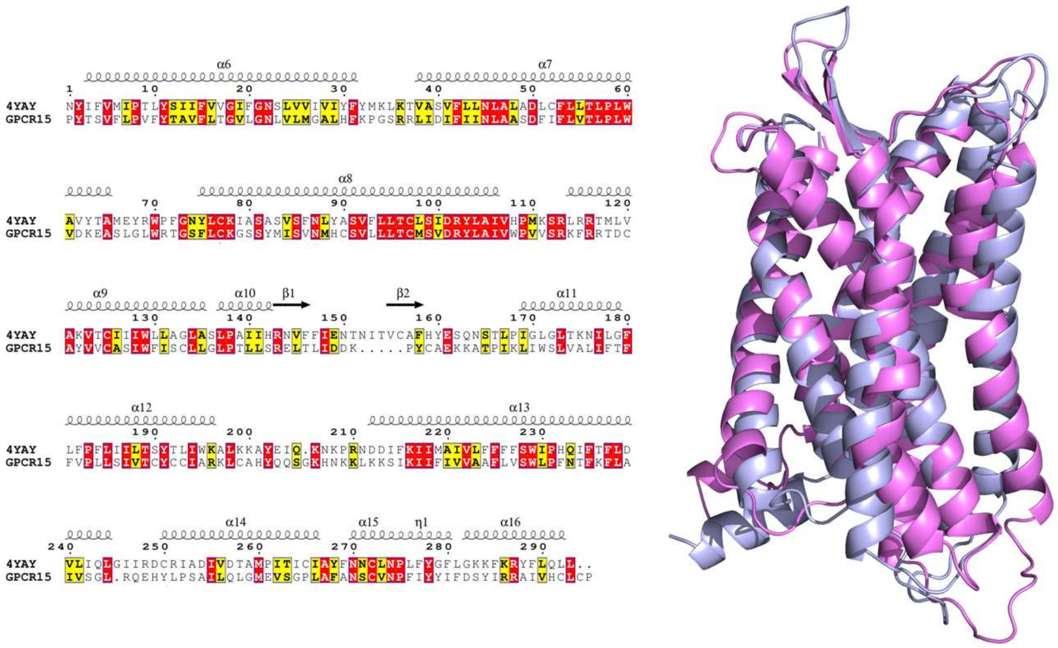

Figure 6. Sequence alignment of the template sequence with the target sequence. The right figure shows the superimposed structure of the homology modeling of GPR15 protein (purple) and template protein (light blue) along with the alignment.

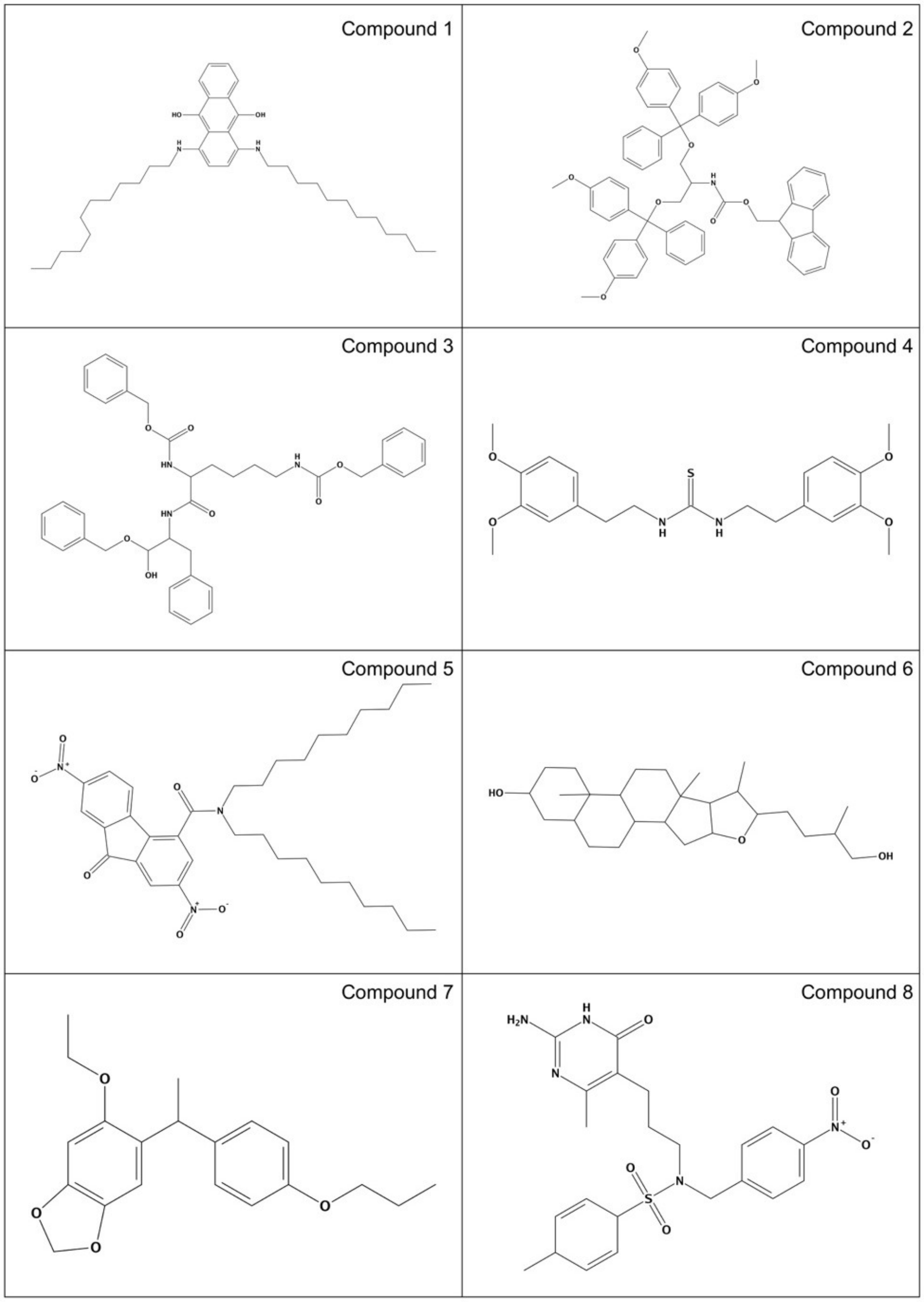

Figure 7. The structure of top 8 hits selected from virtual screening.

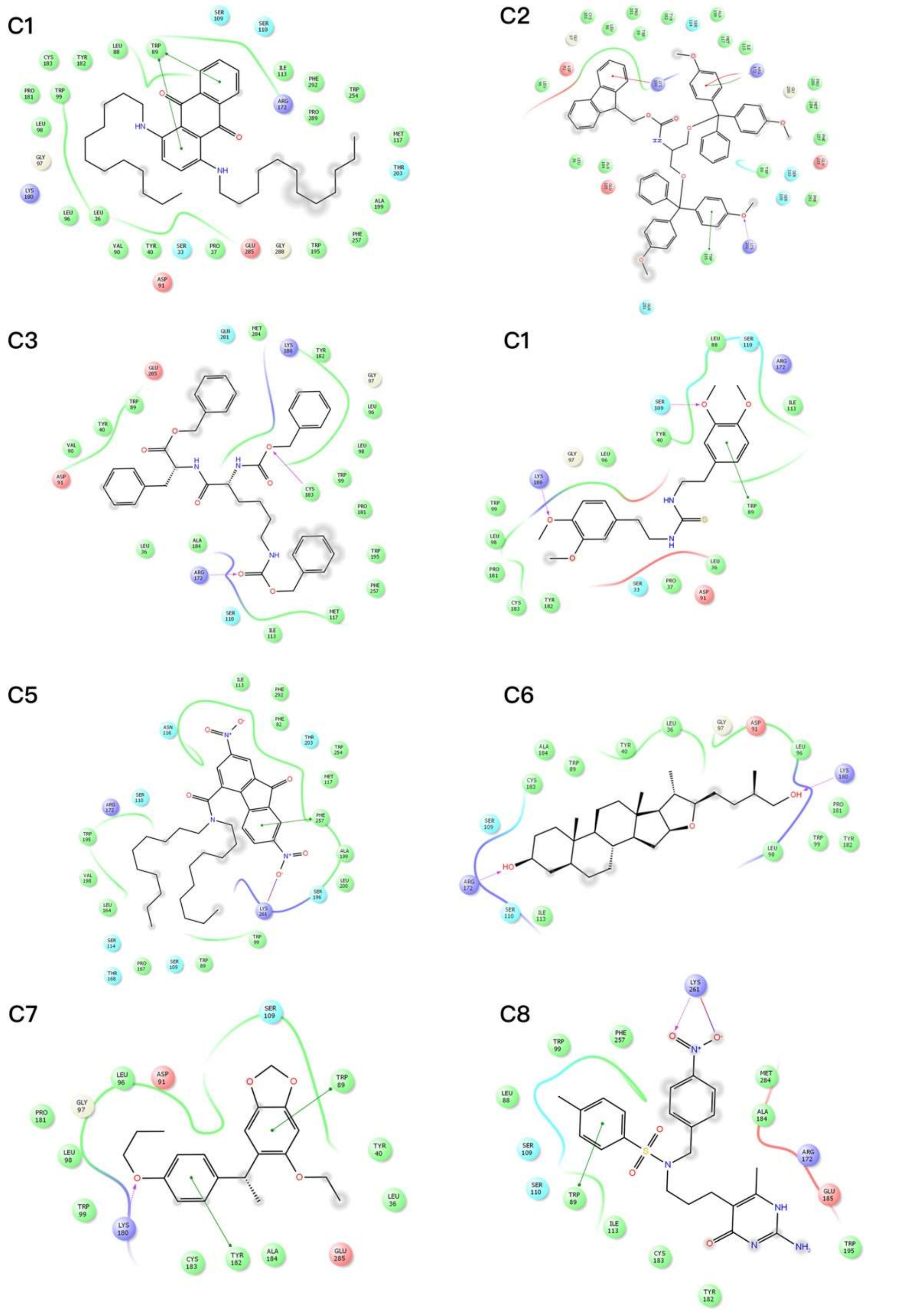

Figure 8. The 2D protein–ligand interactions of representative docking pose of the top five compounds complexed with the GPR15 protein. Hydrogen bonds are illustrated by purple lines, and Pi–pi interactions are marked by green lines.

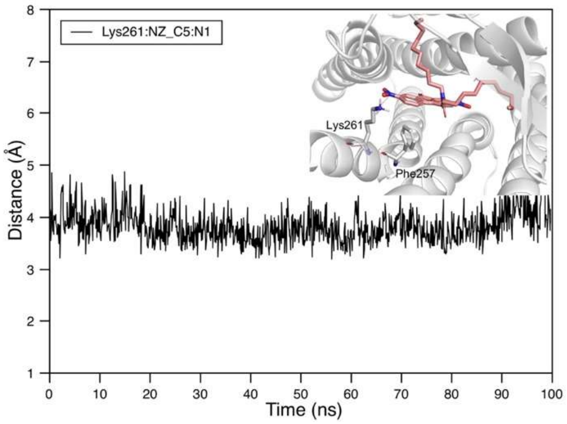

Figure 9. The salt-bridge distance between C5:N1and Lys261:NZ during 100 ns simulations.

Table 1. Top 5 pathways of genes in integrated network.

Pathway identifier	Pathway name	#Entities found	#Entities total	Entities ratio	Entities pValue	Entities FDR	#Reactions found	#Reactions total	Reactions ratio	Submitted entities found
R-HSA-450385	Butyrate Response Factor 1 (BRF1) binds and destabilizes mRNA	2	19	0.00135145	0.00231538	0.24311535	3	6	5.01×10^{-4}	**YWHAB**;EXOSC1
R-HSA-450513	Tristetraprolin (TTP, ZFP36) binds and destabilizes mRNA	2	19	0.00135145	0.00231538	0.24311535	2	4	3.34×10^{-4}	**YWHAB**;EXOSC1
R-HSA-525793	Myogenesis	2	32	0.00227612	0.00636962	0.27046184	3	14	0.00116822 4	MYF6;MEF2D
R-HSA-375170	CDO in myogenesis	2	32	0.00227612	0.00636962	0.27046184	3	14	0.00116822 4	MYF6;MEF2D
R-HSA-388396	GPCR downstream signalling	11	1355	0.09637954	0.00967472	0.27046184	21	253	0.02111148 2	Submitted entities found

Table 2. Top 10 potential cancer types whose prognosis is associated with GPR15. "Rank" stands for the expression abundance rank among all genes.

Cancer	Cox Coefficient	P-Value	FDR Corrected	Rank	Median Expression	Mean Expression
STAD	−0.27	2.20×10^{-3}	1.35×10^{-1}	269	3.95	15.47
HNSC	−0.205	6.40×10^{-3}	1.50×10^{-1}	707	6.15	20.99
LUAD	−0.161	3.90×10^{-2}	1.75×10^{-1}	3711	5.66	15.34
COAD	−0.159	1.50×10^{-1}	5.58×10^{-1}	4299	4.22	15.93
READ	−0.328	1.60×10^{-1}	9.39×10^{-1}	2696	6.83	16.1
LUSC	0.07	3.30×10^{-1}	7.93×10^{-1}	6956	6.93	23.8

KIRC	−0.059	4.80×10^{-1}	6.06×10^{-1}	13174	1.66	7.49
LAML	0.078	5.10×10^{-1}	8.11×10^{-1}	9516	4.88	11.36
ESCA	0.021	8.80×10^{-1}	9.91×10^{-1}	14833	3.06	13.77

Table S3. Correlations between immune infiltrating cells and GPR15 expression level

Cancer	Cell types	Correlation coefficient	P-value	Statistic difference	Correlation strength
COAD	Purity	−0.18383902	1.92×10^{-4}	YES	Very weak
	B Cell	0.280950455	9.16×10^{-9}	YES	Weak
	CD8+ T cell	0.010383547	8.35×10^{-1}	NO	Very weak
	CD4+ T cell	0.292130319	2.38×10^{-9}	YES	Weak
	Macrophage	0.001880939	9.69×10^{-1}	NO	Very weak
	Neutrophil	0.101341327	4.25×10^{-2}	YES	Very weak
	Dendritic Cell	0.149759501	2.61×10^{-3}	YES	Very weak
HNSC	Purity	−0.245063398	3.55×10^{-8}	YES	Weak
	B Cell	0.342491455	1.51×10^{-14}	YES	Weak
	CD8+ T cell	0.394605334	4.12×10^{-19}	YES	Weak
	CD4+ T cell	0.318984967	8.17×10^{-13}	YES	Weak
	Macrophage	0.214987783	1.86×10^{-6}	YES	Weak
	Neutrophil	0.2472179	4.21×10^{-8}	YES	Weak
	Dendritic Cell	0.315589752	1.32×10^{-12}	YES	Weak
LUAD	Purity	−0.314118069	8.96×10^{-13}	YES	Weak
	B Cell	0.247881511	3.28×10^{-8}	YES	Weak
	CD8+ T cell	0.259140634	6.50×10^{-9}	YES	Weak
	CD4+ T cell	0.088275201	5.22×10^{-2}	NO	Very weak
	Macrophage	−0.022504199	6.21×10^{-1}	NO	Very weak
	Neutrophil	0.174530359	1.15×10^{-4}	YES	Very weak
	Dendritic Cell	0.079029988	8.11×10^{-2}	NO	Very weak
STAD	Purity	−0.132792751	9.55×10^{-3}	YES	Very weak
	B Cell	0.24206621	2.55×10^{-6}	YES	Weak
	CD8+ T cell	0.306357949	1.76×10^{-9}	YES	Weak
	CD4+ T cell	0.328503282	1.17×10^{-10}	YES	Weak
	Macrophage	0.224593775	1.29×10^{-5}	YES	Weak
	Neutrophil	0.257657293	4.87×10^{-7}	YES	Weak
	Dendritic Cell	0.284949911	2.32×10^{-8}	YES	Weak

Table 4. The binding free energy (ΔG_{bind} in kcal mol^{-1}) and different energy components for the compound-GPR15 complexes, such as electrostatic energy (ΔG_{ele}), van der Waals (ΔG_{vdW}), and solvation energy (ΔG_{SA} and ΔG_{polar}).

No.	ΔG_{bind}	ΔG_{vdw}	ΔG_{ele}	ΔG_{PA}	ΔG_{SA}
C1	−36.16 ± 0.68	−56.58 ± 0.66	−0.54 ± 0.20	27.87 ± 0.65	−6.90 ± 0.06
C2	−47.64 ± 0.38	−87.90 ± 0.32	−7.79 ± 0.21	57.96 ± 0.42	−9.90 ± 0.02
C3	−36.51 ± 0.39	−71.81 ± 0.40	−11.21 ± 0.30	54.94 ± 0.45	−8.45 ± 0.02
C4	−33.67 ± 0.32	−52.21 ± 0.35	−13.35 ± 0.26	37.15 ± 0.36	−5.25 ± 0.02
C5	−38.88 ± 0.35	−73.81 ± 0.33	−10.39 ± 0.21	53.72 ± 0.31	−8.3 ±0.03
C6	−23.49 ± 0.35	−40.75 ± 0.30	−5.03 ± 0.36	27.56 ± 0.40	−5.29 ± 0.02
C7	−26.76 ± 0.38	−44.73 ± 0.27	−5.63 ± 0.14	28.67 ± 0.26	−5.04 ± 0.01
C8	−18.52 ± 0.38	−47.79 ± 0.24	−8.51 ± 0.26	43.46 ± 0.33	−5.68 ± 0.01

Table 5. Simulation conditions for all systems.

No.	System	Time (ns)	POPC	Ions	Water
1	C1	100	149	24Na^+, 39Cl^-	9277
2	C2	100	149	23Na^+, 38Cl^-	9293
3	C3	100	149	23Na^+, 38Cl^-	9254
4	C4	100	150	24Na^+, 39 Cl^-	9276
5	C5	100	150	24Na^+, 39Cl^-	9306
6	C6	100	149	24Na^+, 39Cl^-	9259
7	C7	100	150	24Na^+, 39Cl^-	9290
8	C8	100	150	24Na^+, 39Cl^-	9263
